# LETTER TO THE EDITOR Usefulness of the Portable DICOM Viewer System for Facial Bone Fractures

**Published:** 2011-12-14

**Authors:** Shimpei Ono, Hiromitsu Hayashi, Junichi Nakao, Takeshi Iimura, Rei Ogawa, Yoshihiro Takami, Hiko Hyakusoku

**Affiliations:** ^a^Departments of Plastic, Reconstructive, and Aesthetic Surgery; ^b^Radiology, Nippon Medical School, Tokyo

Dear Sir,

It is widely recognized that 3-dimensional computed tomography (3D-CT) is useful for diagnosing facial bone fractures.^1^ However, it can be sometimes difficult to obtain such specialized 3D-CT images from radiologists because of the unusual and specific nature of the request. Moreover, it sometimes takes a long time before the 3D photographs are received. Consequently, we have developed a system by which plastic surgeons themselves can immediately and precisely determine the details of a facial fracture. This system involves the use of a personal laptop computer on which a Digital Imaging and Communications (DICOM) viewer has been preinstalled.

This viewer is used as follows. When a patient with suspected facial bone fractures comes to the emergency outpatient clinic, the “volumetric data” of the fractures are captured by the multidetector-row CT with either 8-, 16-, 32-, 64-, or 256-row scanners. The CT images can be reconstructed with the maximum intensity projection and the volume rendering technique. This reconstructed DICOM data can then be analyzed by the plastic surgeon by using the software “OsiriX” (http://www.osirix-viewer.com/), which will work on a personal laptop computer. The author employs a Macintosh OS X for this purpose.

There are several advantages of using this portable DICOM viewer system, which are listed as follows:

1. The plastic surgeon can immediately and precisely determine the details of the facial bone fracture(s) at the initial visit.
2. The plastic surgeon is readily able to focus on the fractured area and can build the 3D images easily (Figure 1).
3. It is not necessary to ask the radiological technicians to perform delicate slice sections (eg, slice width and coronal/sagittal plane sections). It can also eliminate the need to order additional CT scanning if the images are deficient in some way.
4. Because the laptop computer is a portable device, the details of the fractured bone structures can even be readily obtained during surgery.^2^
5. This system allows each orbital floor fracture case with a strangulating inferior rectus muscle to be diagnosed without having to perform an MRI (Figure 2). This helps to speed up the diagnosis of such cases, which can then be rapidly treated by an emergency operation.
6. After one has been familiarized with the system, it is extremely simple to use. It is also very rapid, as it takes about 5 minutes to reconstruct the 3D images.

We retrospectively analyzed 20 orbital fracture patients (average age, 38.5; range, 15-63 years) to whom this portable DICOM viewer system technique was applied, compared to another 20 cases without this technique in Nippon Medical School Hospital in Tokyo.

The average operation time of this technique was compared with that of the standard radiologic approach. In addition, we conducted questionnaire investigations with the 3 surgeons in my department who performed the operations by using this technique. As a result, on average, the actual operation time with this technique was 22% less than the time without this technique. Furthermore, all the 3 surgeons reported that this technique is useful in improving safety and precision in deciding both the size of the bone graft and its site of insertion and in reducing stress during the surgery.

## Figures and Tables

**Figure 1:**
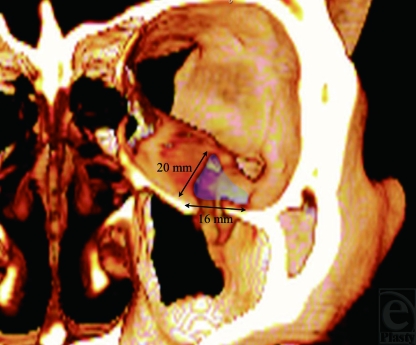
Three-dimensional image showing an orbital floor fracture.

**Figure 2:**
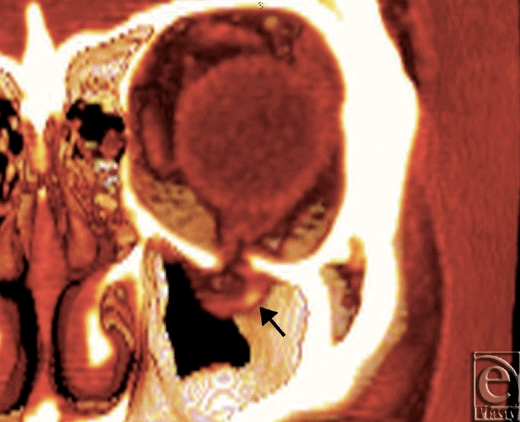
An orbital floor fracture case with a strangulating inferior rectus muscle (black arrow).
